# Recurrent Facial Folliculitis Caused by *Klebsiella aerogenes* Sequence Type 117 in Men who Have Sex with Men

**DOI:** 10.3201/eid3207.260572

**Published:** 2026-07

**Authors:** Gentiane Monsel, Alexandre Bleibtreu, François Durupt, Brigitte Rached, Nicolas Yin, Delphine Martiny, Jonathan Krygier, Olivier Chosidow, Landon Porter, Matthieu Godinot, Erwan Turquier, Estelle Hau, Jean-Noël Dauendorffer, Maxime Bonjour, Marie Jachiet, Mathilde Liberge, Béatrice Berçot, Valérie Pourcher, Vincent Bérot, Marion Levast, Olivier Dauwalder, Romain Salle, Sébastien Fouéré, Cécile Brin, Laurent Dortet, Cécile Emeraud

**Affiliations:** Pitié Salpêtrière Hospital, APHP, Paris, France (G. Monsel, A. Bleibtreu, B. Rached, E. Turquier, V. Pourcher, V. Bérot); Dermatologic Infectiology and Sexually Transmitted Infections Working Group of the French Society of Dermatology, Paris (G. Monsel, F. Durupt, J. Krygier, O. Chosidow, J.-N. Dauendorffer, M. Jachiet, V. Bérot, R. Salle, S. Fouéré, C. Brin); Croix Rousse Hospital, Hospices Civils de Lyon, Lyon, France (F. Durupt, M. Godinot, M. Bonjour, O. Dauwalder); Laboratoire Hospitalier Universitaire de Bruxelles–Universitair Laboratorium Brussel, Université Libre de Bruxelles, Brussels, Belgium (N. Yin, D. Martiny); University of Mons, Mons, Belgium (D. Martiny); St. Pierre University Hospital, Université Libre de Bruxelles, Brussels (J. Krygier); Ambroise Paré-UVSQ and Bicêtre-Paris Saclay Hospitals, APHP, Paris (O. Chosidow); University of Texas at Austin, Austin, Texas, USA (L. Porter); Edouard Herriot Hospital, Hospices Civils de Lyon, Lyon (M. Godinot); Saint Louis Hospital, Paris Cité University, APHP, Paris (E. Hau, J.-N. Dauendorffer, M. Jachiet, M. Liberge, B. Berçot, S. Fouéré); Sorbonne University, INSERM UMR S 1136, Pierre Louis Epidemiology and Public Health Institute, Paris (V. Pourcher); Savoie Metropole Hospital, Chambéry, France (M. Levast, C. Brin); Centre National de Référence des Staphylocoques, Lyon (O. Dauwalder); Centre for Genital and Sexually Transmitted Diseases, Hôtel-Dieu Hospital, APHP, Paris (R. Salle); Kremlin Bicêtre Hospital, APHP, Le Kremlin Bicêtre, France (L. Dortet, C. Emeraud); Associated French National Reference Center for Antibiotic Resistance: Carbapenemase-Producing Enterobacteriaceae, Le Kremlin-Bicêtre (L. Dortet, C. Emeraud); IHU SEPSIS Comprehensive Sepsis Center, Université Paris-Saclay, Le Kremlin Bicêtre (L. Dortet, C. Emeraud)

**Keywords:** Klebsiella aerogenes, folliculitis, bacteria, men who have sex with men, MSM, sexually transmitted infections, France, Belgium, United States

## Abstract

We describe 17 cases of recurrent facial folliculitis caused by *Klebsiella aerogenes* bacteria in men who have sex with men in France, Belgium, and the United States. Whole-genome sequencing showed all isolates belonged to sequence type 117 or related lineages. Our findings suggest sexual transmission and highlight emerging clinical and public health concerns.

*Klebsiella aerogenes* (formerly *Enterobacter aerogenes*) is a gram-negative bacillus present in the environment and human gut microbiota and is an uncommon cause of community-acquired skin and soft tissue infections. Since 2025, a pattern of relapsing facial folliculitis caused by *K. aerogenes* has been reported in France, Belgium, and Spain (*1*–*3*). Those cases occurred exclusively among men who have sex with men (MSM), which strongly suggests that *K. aerogenes* could be an emerging sexually transmissible pathogen within that population (*1*–*3*). Whole-genome sequencing (WGS) of 4 isolates from Belgium revealed they all belonged to sequence type (ST) 117 (*2*), suggesting the emergence of a particular clone. Unfortunately, isolates reported in France and Spain were not sequenced, precluding confirmation of that hypothesis (*1*,*3*). We describe a multinational case series of 17 MSM with recurrent *K. aerogenes* facial folliculitis (KAFF) and analyze their epidemiologic, clinical, microbiologic, and genomic characteristics to assess the emergence of a specific lineage.

## The Study

We retrospectively identified cases through clinician notification within the Dermatologic Infectiology and Sexually Transmitted Infections Working Group in Paris, Lyon, and Chambéry, France; Brussels, Belgium; and Austin, Texas, USA. We extracted clinical data from medical records. We defined disease duration as the time from symptom onset to healing or last follow-up. We recovered 24 *K. aerogenes* isolates from 17 patients, with multiple isolates in cases of recurrence or longitudinal sampling.

**Figure 1:**
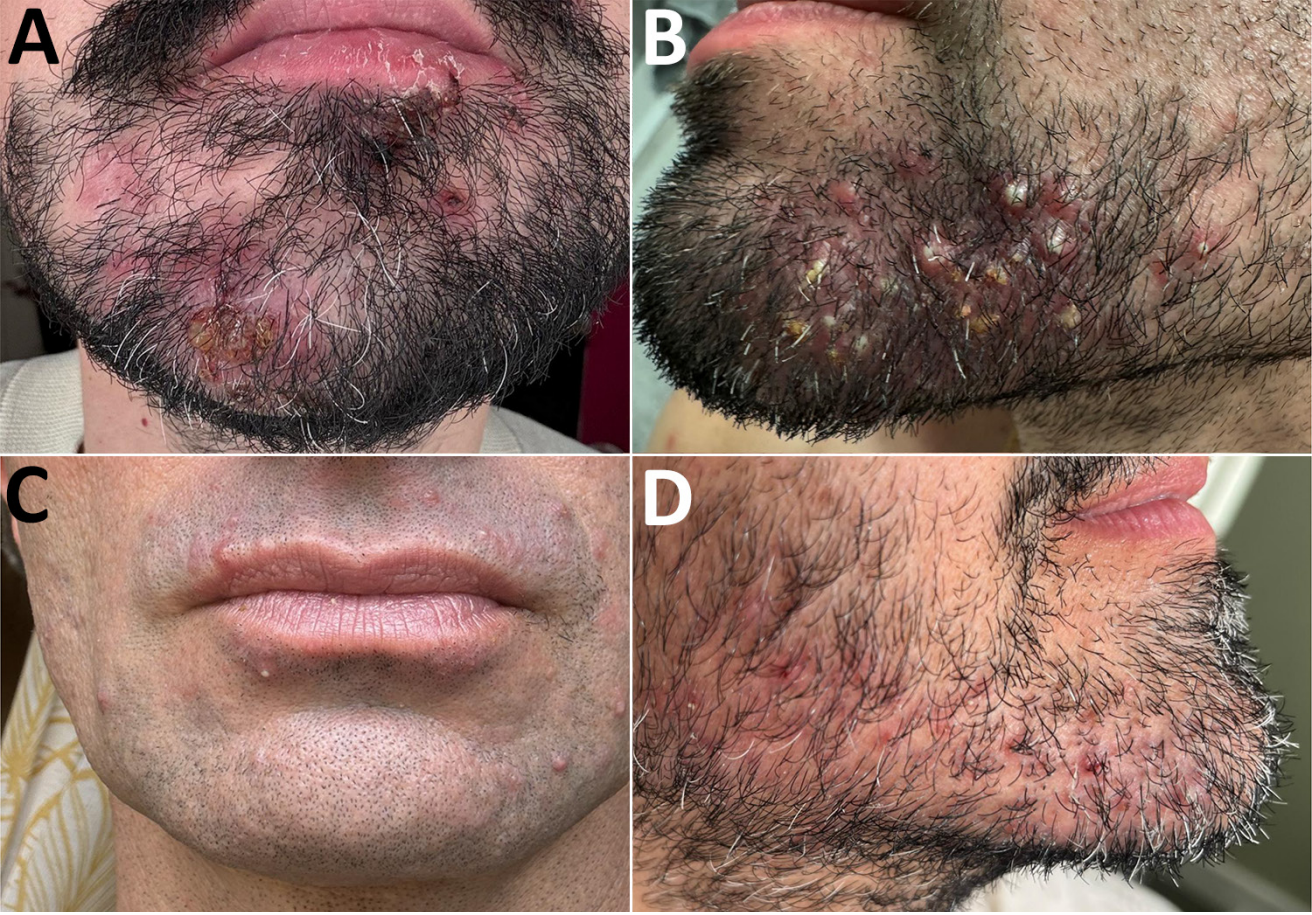
Clinical appearance of patients in study of *Klebsiella aerogenes* sequence type 117 facial folliculitis in men who have sex with men in France, Belgium, and the United States. A) Beard sycosis with painful purulent lesions (deep folliculitis). B) Papulopustular lesions in the beard region, consistent with both superficial and deep folliculitis. C) Pustular lesions of the mustache and beard area (superficial folliculitis). D) Multiple erythematous papules and pustules involving the right cheek and chin (superficial folliculitis).

All 17 patients were MSM; median age was 35 years (interquartile range [IQR] 31.5–45.5 years) (Appendix 1 Table). None of the patients were HIV-positive or immunocompromised; 9 were receiving HIV preexposure prophylaxis (PrEP). Ten patients reported history of sexually transmitted infections (STIs); 11 had a remote history (>10 years earlier) of acne treated with isotretinoin, doxycycline, or both, followed by a prolonged symptom-free interval before the folliculitis. Eight patients reported regular attendance at communal facilities (hot tubs, saunas, swimming pools, or gyms). We classified lesions as superficial for follicular pustules or inflammatory papules, deep for painful purulent nodules indicating deeper follicular involvement, or mixed. Lesions were exclusively facial, predominantly in the beard and mustache areas (Figure 1).

The median time from lesion onset to diagnosis was 7 (IQR 1.5–16) months. Before *K. aerogenes* identification, patients received antimicrobial drugs targeting gram-positive cocci without improvement. In recurrent or persistent cases, repeated cultures consistently yielded *K. aerogenes*. Nasal swab cultures were positive in 8 (73%) of 11 tested patients. Mycologic samples remained negative, thereby excluding tinea barbae. Median disease duration was 23 (IQR 9–54) months. Thirteen patients initially responded to targeted antimicrobial therapy but relapsed within 1–3 weeks after discontinuation. Overall, 12 patients did not achieve cure, 2 achieved sustained remission, 2 were still under treatment, and 1 did not complete follow-up care.

We performed WGS on all 24 isolates using short-read technology (Hiseq; Illumina, https://www.illumina.com). We conducted multilocus sequence typing (MLST) and resistome analysis using PubMLST (https://pubmlst.org) and ResFinder (https://genepi.food.dtu.dk/resfinder) databases. All folliculitis isolates belonged to ST117, except 1 ST117-like isolate (1FOLC4) with 1 nucleotide variation in *rplB*. One isolate (1FOLB1) acquired the *sul2* gene, conferring sulfamethoxazole resistance, which was absent in earlier isolates from the same patient; no other acquired resistance genes were detected.

**Figure 2:**
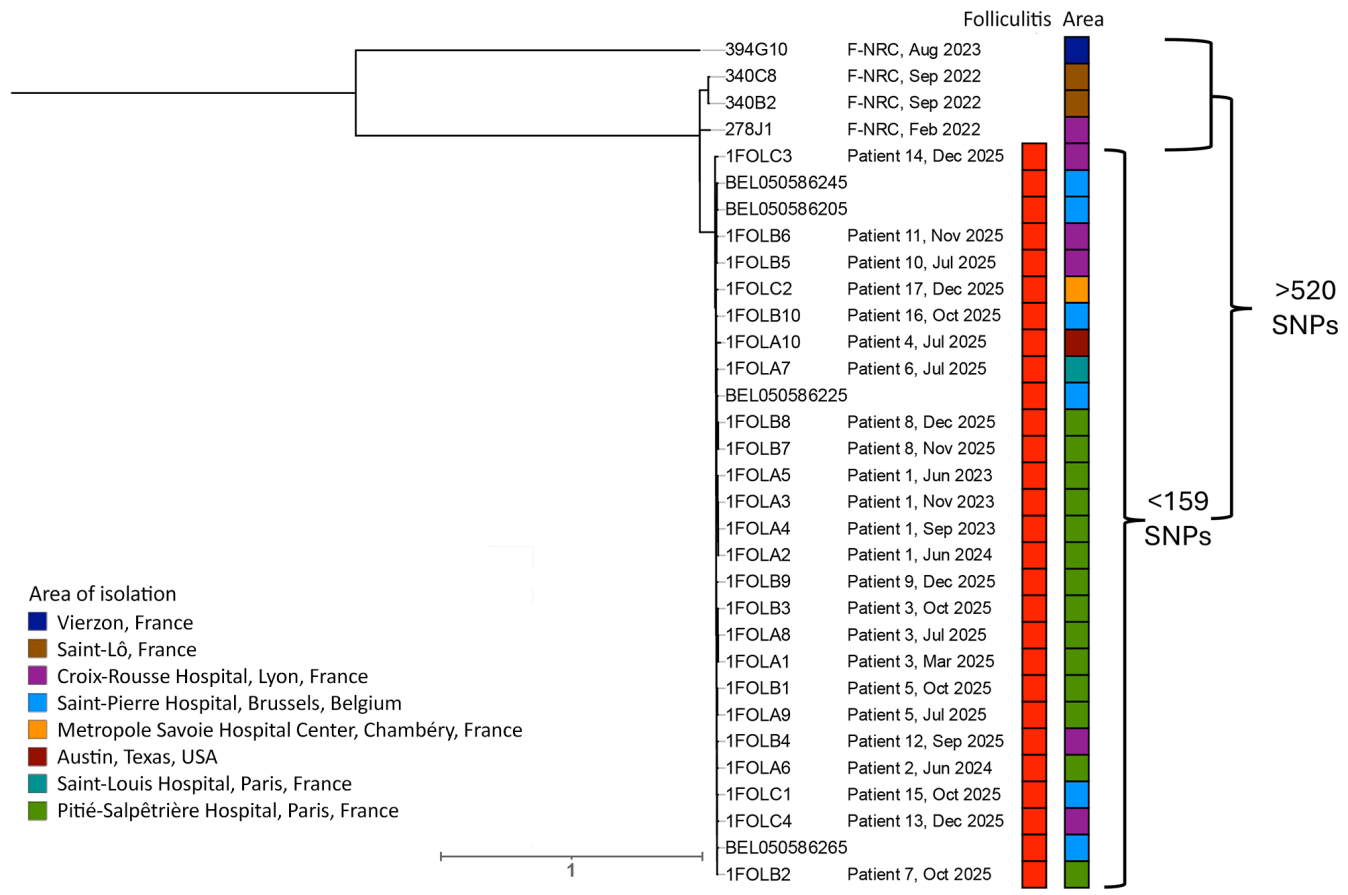
SNP-based phylogeny of *Klebsiella aerogenes* sequence type (ST) 117 and related isolates from study of facial folliculitis in men who have sex with men in France, Belgium, and the United States. Phylogenetic tree shows 24 folliculitis-associated isolates from 17 patients, 4 unrelated ST117 isolates from the French National Reference Center not implicated in folliculitis, and 4 ST117 folliculitis isolates collected in Belgium in 2025 (*2*). An expanded version of this figure is available online (https://wwwnc.cdc.gov/EID/article/32/7/26-0572-App2.pdf). F-NRC, French National Reference Center; SNP, single-nucleotide polymorphism.

We assessed genetic diversity and relatedness phylogenetic analysis using SNIppy version 4.6.0 (https://github.com/tseemann/snippy); reference genome was strain 1FOLA5. Analysis included 4 additional *K. aerogenes* ST117 isolates from the French National Reference Center for carbapenem resistance, which were unrelated to folliculitis cases and genomes from a previous study (*2*). Folliculitis-associated isolates were highly divergent from unrelated ST117 isolates (>520 single-nucleotide polymorphisms [SNPs]) but clustered together regardless of geographic origin (Figure 2; Appendix 2 Figure). Within that cluster, pairwise distances were 1–159 SNPs; for most isolates they were 60−80 SNPs (Figure 2; Appendix 2 Figure). Given an estimated evolutionary rate of 7–10 SNPs per year in Enterobacterales (*4*), those observed distances suggest a recent common ancestor and support the recent emergence and international dissemination of this lineage within interconnected MSM sexual networks. 

## Conclusions

Although *K. aerogenes* is not considered a sexually transmitted pathogen, our findings support transmission through intimate contact: all cases occurred in MSM, many patients had previous STIs or were receiving PrEP, and the distribution of lesions is compatible with exposure during oro-anal contact. However, direct evidence of sexual transmission is lacking, no contact tracing was conducted, no documented transmission events were identified, and alternative routes including shared environmental exposures cannot be excluded. The emergence and intercontinental dissemination of enteric pathogens in MSM, such as *Shigella* spp., *Campylobacter jejuni*, and extended-spectrum β-lactamase–producing Enterobacterales, have been well documented (*5*–*7*). Rectal swab specimens were collected from some patients to assess potential enteric carriage, but *K. aerogenes* was not identified within the fecal Enterobacterales flora. Similarly, sexually transmissible dermatophytes, such as *Trichophyton mentagrophytes* internal transcribed spacer genotype VII, as well as monkeypox virus, illustrate the expanding spectrum of pathogens transmitted through intimate contact (*8*,*9*). Because half of the patients in this investigation reported exposure to communal water facilities, nonsexual transmission may also occur. The condition might be better classified as sexually transmissible, rather than strictly sexually transmitted (*10*).

KAFF appears to be an uncommon and likely underrecognized condition, consistent with previous reports of gram-negative folliculitis in patients who had acne after prolonged antibiotic exposure (*11*,*12*). Because *K. aerogenes* is not a recognized skin pathogen, it may be overlooked in cutaneous samples, which contributes to underdiagnosis. The broad clinical spectrum, from superficial to disfiguring folliculitis, suggests a major role for host factors (*11*). Its strict facial localization points to regional determinants such as sebum composition and the follicular microenvironment (*13*). Previous use of systemic antimicrobial drugs can disrupt the cutaneous and nasal microbiome, reducing colonization resistance and promoting the emergence of gram-negative organisms such as *K. aerogenes* (*2*,*11*–*13*).

We observed nasal colonization consistent with previous descriptions of gram-negative folliculitis (*11*,*12*), supporting the hypothesis that the nasal cavity is a reservoir for *K. aerogenes* (*2*,*3*,*12*). However, its clinical significance and role in recurrence remain unclear. A study in Guangzhou, China, reported nasal carriage rates of ≈30% among MSM; that study identified 2 ST117 strains, supporting international dissemination of that lineage (*14*). In several patients, relapses occurred despite elimination of predisposing factors, suggesting persistence in a difficult-to-eradicate reservoir (*1*). Dysbiosis of skin microbiota and bacterial factors such as virulence traits or biofilm formation might also contribute to persistence and treatment failure (*13*,*14*,*15*).

Therapeutic management of KAFF remains challenging (*1*–*3*). Although trimethoprim/sulfamethoxazole or fluoroquinolones often lead to apparent clinical cure, relapses commonly occur soon after treatment discontinuation (*1*–*3*). Those recurrences have prompted consideration of nonantimicrobial approaches such as oral isotretinoin (*1*,*3*), which might act by reducing sebum production and follicular reservoirs rather than through direct antibacterial activity (*12*). By limiting repeated antimicrobial exposure and associated cutaneous dysbiosis, isotretinoin could help restore microbial balance (*13*), although its mechanisms and clinical effectiveness in KAFF remain uncertain. The emergence of the recurrent treatment-refractory ST117 clone in MSM is particularly concerning in the context of increasing antimicrobial pressure from routine STI management and implementation of doxycycline postexposure prophylaxis.

Our observations highlight the need for further research into the virulence determinants of *K. aerogenes* ST117, its reservoirs, and its modes of transmission. Studies integrating clinical data, microbiology, and microbiome analyses will be crucial to clarify the role of host factors, antimicrobial exposure, and skin ecosystem disruption in the pathogenesis and persistence of that pathogen and determine the optimal therapeutic strategy. Surveillance in sexual health clinics and dermatology care will better define the burden of KAFF and guide prevention strategies.

Appendix 1 Case data from study of recurring facial folliculitis in men who have sex with men.

Appendix 2 Additional phylogenetic information from study of recurring facial folliculitis in men who have sex with men.
