# Supplementary material for: Recurrent Facial Folliculitis Caused by Klebsiella aerogenes Sequence Type 117 in Men who Have Sex with Men
Source: Emerg Infect Dis. 2026 Jul;32(7):1172–6. doi: 10.3201/eid3207.260572 (PMC13322422; doi:10.3201/eid3207.260572)
Supplement: Appendix 2 — Additional phylogenetic information from study of recurring facial folliculitis in men who have sex with men. [file 26-0572-Techapp-s2.pdf]

Article DOI: https://doi.org/10.3201/eid3207.260572

EID cannot ensure accessibility for supplementary materials supplied by authors. Readers who have difficulty accessing supplementary content should contact the authors for assistance.

Recurrent Facial Folliculitis Caused by *Klebsiella aerogenes* Sequence Type 117 in Men who Have Sex with Men

Appendix 2

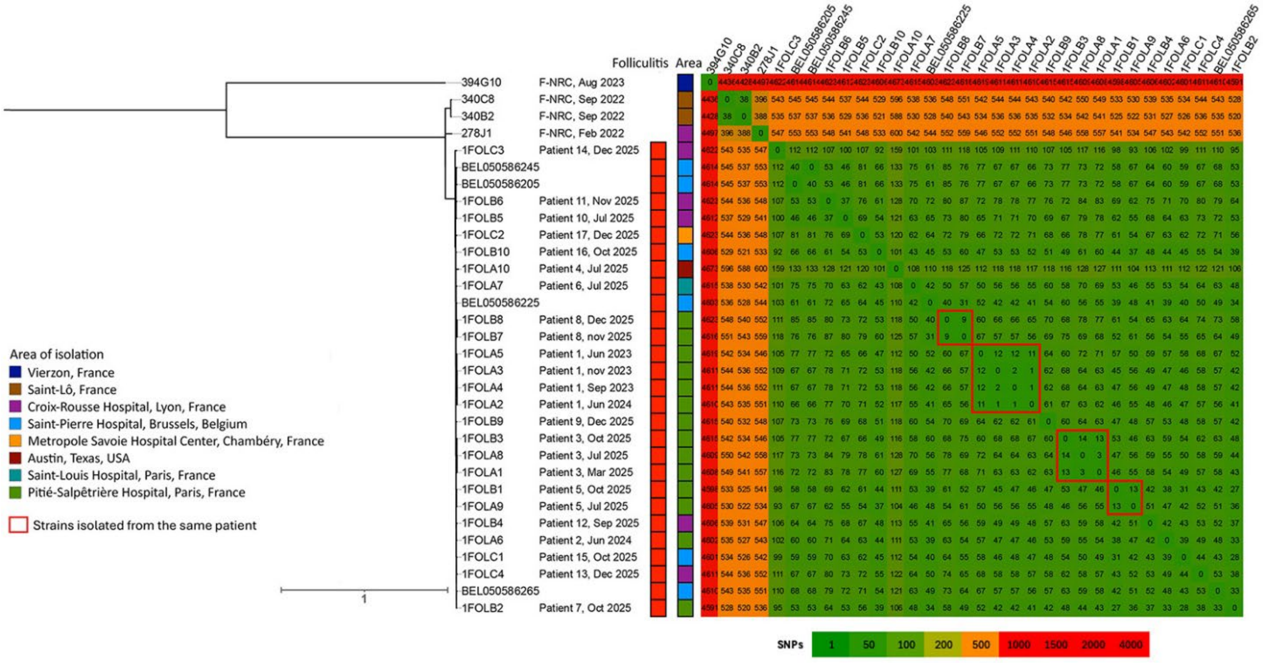

Appendix 2 Figure. SNP-based phylogeny of *Klebsiella aerogenes* ST117 and related isolates. Phylogenetic tree showing 24 folliculitis-associated isolates from 17 patients, 4 unrelated ST117 isolates from the French National Reference Center not implicated in folliculitis, and 4 ST117 folliculitis isolates collected in Belgium in 2025 (*2*). Boxed isolates indicate those recovered from the same patient.
